# Biodegradable Magnesium Bone Implants Coated with a Novel Bioceramic Nanocomposite

**DOI:** 10.3390/ma13061315

**Published:** 2020-03-13

**Authors:** Mehdi Razavi, Mohammadhossein Fathi, Omid Savabi, Lobat Tayebi, Daryoosh Vashaee

**Affiliations:** 1 Biionix^TM^ (Bionic Materials, Implants & Interfaces) Cluster, Department of Internal Medicine, College of Medicine, University of Central Florida, Orlando, FL 32827, USA; 2 Department of Materials Science & Engineering, University of Central Florida, Orlando, FL 32816, USA; 3 Biomaterials Research Group, Department of Materials Engineering, Isfahan University of Technology, Isfahan 84156-83111, Iran; fathi@cc.iut.ac.ir; 4 Dental Materials Research Center, Isfahan University of Medical Sciences, Isfahan 81746-73461, Iran; 5 Torabinejad Dental Research Center, School of Dentistry, Isfahan University of Medical Sciences, Isfahan 81746-73461, Iran; savabi@dnt.mui.ac.ir; 6 Marquette University School of Dentistry, Milwaukee, WI 53233, USA; lobat.tayebi@marquette.edu; 7 Electrical and Computer Engineering Department, North Carolina State University, Raleigh, NC 27606, USA; 8 Materials Science and Engineering Department, North Carolina State University, Raleigh, NC 27606, USA

**Keywords:** biodegradable magnesium implants, bioceramics, corrosion, bioactivity, orthopedic implant

## Abstract

Magnesium (Mg) alloys are being investigated as a biodegradable metallic biomaterial because of their mechanical property profile, which is similar to the human bone. However, implants based on Mg alloys are corroded quickly in the body before the bone fracture is fully healed. Therefore, we aimed to reduce the corrosion rate of Mg using a double protective layer. We used a magnesium-aluminum-zinc alloy (AZ91) and treated its surface with micro-arc oxidation (MAO) technique to first form an intermediate layer. Next, a bioceramic nanocomposite composed of diopside, bredigite, and fluoridated hydroxyapatite (FHA) was coated on the surface of MAO treated AZ91 using the electrophoretic deposition (EPD) technique. Our in vivo results showed a significant enhancement in the bioactivity of the nanocomposite coated AZ91 implant compared to the uncoated control implant. Implantation of the uncoated AZ91 caused a significant release of hydrogen bubbles around the implant, which was reduced when the nanocomposite coated implants were used. Using histology, this reduction in the corrosion rate of the coated implants resulted in an improved new bone formation and reduced inflammation in the interface of the implants and the surrounding tissue. Hence, our strategy using a MAO/EPD of a bioceramic nanocomposite coating (i.e., diopside-bredigite-FHA) can significantly reduce the corrosion rate and improve the bioactivity of the biodegradable AZ91 Mg implant.

## 1. Introduction

Over the last decade, the development of biodegradable orthopedic implants has significantly advanced [1,2,3]. Completed and ongoing clinical trials for bone repair and regeneration are mostly focused on biodegradable ceramics include calcium phosphate (ClinicalTrials.gov Identifier: NCT02153372, and NCT02803177 in Germany). Although calcium phosphates are known to be bioactive and support osteoblast adhesion and proliferation [4,5], their major limitation is mechanical properties; namely, they are brittle with a poor fatigue resistance [6,7,8]. Brittleness so far restricted their application to non-load bearing areas, filler or coating [9], rendering it impossible to use them for the repair or regeneration of load-bearing bone defects [10,11,12]. Biodegradable polymers such as poly-L-lactic acid, poly-glycolic acid, and copolymers with Food and Drug Administration (FDA) approval for human clinical use have also been extensively used in preclinical studies of bone tissue engineering. However, the application of biopolymer materials suffers from poor processability and weak mechanical properties [13]. Magnesium (Mg) alloys are significantly more flexible than bioceramics, mechanically stronger than biopolymers with the advantage of bioabsorption capabilities over other biometals [14,15,16]. Mg is an essential mineral crucial to bone health and can even stimulate new bone formation, and its physical and mechanical properties are similar to those of human bones [14,17,18]. Given these characteristics, Mg is considered an attractive element for forming a bone implant. However, a series of clinical trials using Mg-based implants failed prematurely due to the Mg’s rapid corrosion and high hydrogen-evolution [14]. A strategy to tackle this issue is to reduce Mg’s corrosion rate [19], and the promising future of biodegradable Mg implants is dependent on being able to reduce their corrosion. Several treatments have been proposed to reduce Mg’s corrosion rate, including Mg purification [20], fluoride conversion coatings [21], alloying [19], anodizing [22], and compositing [23].

An effective technique to reduce the Mg’s corrosion rate is surface coating [24]. For a bone implant, the surface coating can also enhance the implant’s surface bioactivity (i.e., osteoproductivity), thereby resulting in improved bone-implant integration [25]. As a coating material, silicate glass-ceramics are a suitable option given their low biodegradation and their ability for new bone formation [26,27]. Biocompatible silicate glass-ceramics are diopside (CaMgSi_2_O_6_) [28], akermanite (Ca_2_MgSi_2_O_7_) [29], merwinite (Ca_3_MgSi_2_O_8_) [30], and bredigite (Ca_7_MgSi_4_O_16_) [31]. We have previously synthesized and separately coated the mentioned glass-ceramics on Mg implants [29,32,33,34]. Our results showed that, among tested glass-ceramics, bredigite had the least biodegradation, and diopside indicated the greatest bioactivity.

Furthermore, compared to calcium phosphates coated on the surface of Mg implants, fluoridated hydroxyapatite (FHA: Ca_10_(PO_4_)_6_(OH)_2-x_F_x_) has indicated the improved bioactivity and biocompatibility [35]. Accordingly, we synthesized a diopside-bredigite-fluoridated hydroxyapatite nanocomposite and coated on the surface of an Mg implant. We used the electrophoretic deposition (EPD) method to coat our nanocomposite on the surface of the AZ91 Mg alloy. EPD was chosen since it offers many advantages as a coating method, including simplicity, cost-effectiveness, and environmentally friendly processing [36,37]. EPD has also already been utilized for coating the bioceramics on the surface of biometals for orthopedic implants [38,39,40]. However, before coating with EPD, we first treated our AZ91 substrate with micro-arc oxidation (MAO) technique. On an Mg alloy, a conversion coating such as MAO acts as an intermediate layer to reduce not only the Mg’s corrosion rate, but also enhance the adhesion between the Mg and final coating [22].

Hence, the main aim of this work was to reduce the corrosion rate and also enhance the bioactivity of a biodegradable AZ91 Mg implant using a nanocomposite coating composed of diopside, bredigite, and fluoridated hydroxyapatite which was prepared using MAO/EPD technique.

## 2. Materials and Methods

### 2.1. Preparation of AZ91 Mg Alloy Substrate

A commercial AZ91 Mg alloy (Al 9%, Zn 1%, Mn 0.2%, Fe < 0.005%, all in wt.%) was machined to obtain the substrates with dimensions of 20 × 15 × 5 mm. Samples were then polished with SiC papers from 80 to 600 grit.

### 2.2. Surface Coating

#### 2.2.1. Nanocomposite Powder Preparation

The diopside, bredigite, and fluoridated hydroxyapatite (FHA) nanoparticles were first separately synthesized according to our previously published protocols [16,41,42]. They were then blended with the ratio of 1/3:1/3:1/3, respectively, to acquire the nanocomposite powder. The nanocomposite powder was then coated on the surface of AZ91 samples using the combined MAO/EPD method.

#### 2.2.2. Micro Arc Oxidation (MAO)

MAO was performed according to our previously published protocol [22]. In brief, an AZ91 sample was used as the anode and a stainless-steel plate as the cathode electrode, an aqueous solution of NaOH (200 g/L) and Na_2_SiO_3_ (200 g/L) as the electrolyte along with a power supply. MAO treatment was performed in the applied voltage of 60 V for 30 min.

### 2.3. Electrophoretic Deposition (EPD)

**Figure 1:**
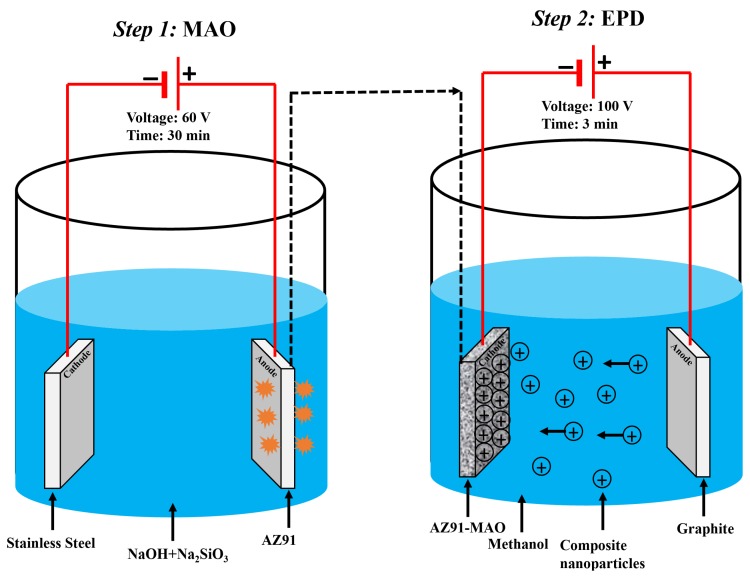
**Schematic representation of MAO-EPD coating method:** In step 1, i.e., MAO, a power supply was used; an AZ91 sample was used as the anode and a stainless-steel plate as the cathode electrodes; a mixture of NaOH (200 g/L) and Na_2_SiO_3_ (200 g/L) were also used as the electrolyte solution. MAO was performed in the voltage of 60 V for 30 min. In step 2, i.e., EPD, an AZ91 sample was used as the cathode and a graphite rod as the anode electrode. A suspension of nanocomposite particles at a concentration of 10 g particles/100 mL methanol was also used as the electrolyte. EPD was then performed under a voltage of 100 V for 3 min at room temperature.

EPD was performed according to our previously published protocol [42]. In brief, an AZ91 sample was used as the cathode and a graphite rod as the anode electrode. The electrolyte was a suspension of nanocomposite particles at a concentration of 10 g particles/100 mL methanol (99.9%). Before starting the EPD process, the nanoparticles were dispersed into the suspension by placing them in an ultrasonic bath for 60 min and then stirring with a magnetic stirrer for 30 min. EPD was then performed under a constant voltage of 100 V for 3 min at room temperature (Figure 1).

### 2.4. Characterizations

The size and morphology of synthesized nanocomposite particles were measured using a transmission electron microscope (TEM, JEOL JEM-2100). The morphology and chemical composition of surfaces were observed under a scanning electron microscope (SEM) equipped with energy dispersive spectroscopy (EDS) (Philips XL 30: Eindhoven). The topography of surfaces was also observed using a laser scanning microscope (LSM) (Keyence, VK X100/X200). Phase structure analysis was performed using an X-ray diffractometer (XRD, Philips Xpert). The obtained XRD patterns were compared with the standards compiled by the Joint Committee on Diffraction Pattern and Standards (JCDPS). The grain size of synthesized nanocomposite particles was also estimated by broadening XRD peaks using the Williamson–Hall equation (Equation (1)) [43]:

β cosθ = 0.89 λ/D + 2ε sinθ(1)

where β is the full width of diffraction peak (rad) in the middle of its height, θ is Bragg’s angle (°), and λ is the wavelength of the X-ray (nm) considered after computer fitting of the X-ray data using Gaussian line shape. When β cosθ is plotted against sinθ, a straight line is obtained with the slope of 2ε and the intercept as (0.89 λ/D) and the grain size, d (nm), can be calculated.

A compression test was performed on our AZ91 Mg alloy according to ASTM E9 standard. The rod samples with a diameter of 3 mm and a length of 6 mm were machined for the experiment. To measure the compressive properties of samples, we used an INSTRON 8562 universal tensile testing machine at a crosshead displacement rate of 0.5 mm/min.

### 2.5. Corrosion Tests

#### 2.5.1. Electrochemical Test

A PARSTAT 2273 Ametek potentiostat was used for measuring the electrochemical (i.e., polarization and electrochemical impedance spectroscopy (EIS)) properties of samples in the standard simulated body fluid (SBF) solution prepared according to Kokubo’s protocol [44]. A three-electrode cell was used include the working electrode (i.e., AZ91 sample), the reference electrode (i.e., calomel), and the counter electrode (i.e., platinum). The experiment started after the sample was incubated in the SBF solution for 60 min to be stabilized, and a scanning rate of 1 mV.s^−1^ was applied for the polarization experiment. The impedance data were recorded with a frequency range of 100 kHz to 10 mHz.

#### 2.5.2. Immersion Test

The immersion test was performed in the SBF, according to ASTM-G31-72 [45], to monitor the corrosion rate of samples. Each sample was individually immersed into a falcon tube containing SBF and incubated at 37 °C for 672 h (28 days). The corrosion rate was determined by measuring the weight loss of each sample after 0, 72 h, 168 h, 336 h, 504 h, and 672 h immersion. The corrosion products formed on the surface of samples during the corrosion were removed using chromic acid (200 g/L CrO_3_) [16]. The difference in weight of samples before and after the immersion into chromic acid showed the amount of weight loss, and the corrosion rate of the samples was then calculated using the weight loss as a function of immersion time, according to Equation (2):

Corrosion rate = W/At(2)

where W is the weight loss, A is the sample’s surface area exposed to the SBF, and t is the immersion time.

### 2.6. In Vivo Animal Study

The animal experiments in our study were approved by the University Ethics Committee of the Isfahan University of Medical Sciences. Rabbits with average weights of 3 kg were anesthetized by subcutaneous administration of Ketamine (35 mg/kg), Xylazine (5 mg/kg) and Acepromazine (1 mg/kg). After anesthesia, the operation sites were shaved, decortication was carried out, and then the holes with 3 mm diameter were created at the greater trochanter of rabbits using a hand driller. AZ91, MAO, and composite coated rod implants (n = 3) were then implanted into the created bone defects. After the operation, all rabbits received subcutaneous injections of antibiotics. The rabbits were then allowed to move freely in their cages without any external support. The rabbits were sacrificed 2 months post-implantation. Also, a radiography imaging was performed on the implantation site 2 weeks after the surgery and prior to sacrificing the rabbits. For histology, the bone samples were fixed in the formaldehyde solution (4%), dehydrated using ethanol, and then decalcified in a nitric acid solution. The samples were then embedded in paraffin and cut into films. The sectioned samples were stained with Hematoxylin and Eosin (H&E) stain, and the morphological and histological analyses were performed under a microscope to observe the bone regeneration and inflammation around the implant.

### 2.7. Statistical Analysis

All values were expressed as the mean ± standard deviation (SD). Statistical analysis of all quantitative data was performed using a one-way ANOVA (Analysis of Variance) with post-hoc Tukey test (Astatsa.com; Online Web Statistical Calculators, USA) with any differences considered statistically significant when *p* < 0.05.

## 3. Results and Discussion

### 3.1. Characterizations

**Figure 2:**
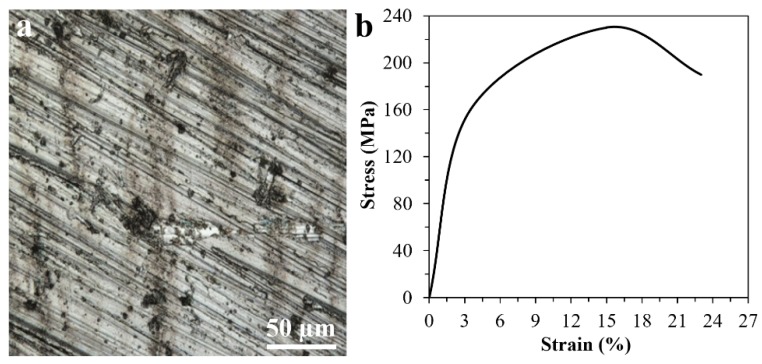
LSM image (**a**) and compressive stress-strain curve (**b**) of our AZ91 Mg alloy.

The microstructure of our AZ91 Mg alloy substrate has been shown in Figure 2a. Using compression test, we determined that this alloy has an elastic modulus of 45 GPa (Figure 2b), which is similar to human cancellous bones (40 GPa) [46]. This lower elastic modulus compared to biometals or bioceramics enhances implant-to-bone stress loading and can minimize bone atrophy due to stress shielding [47].

**Figure 3:**
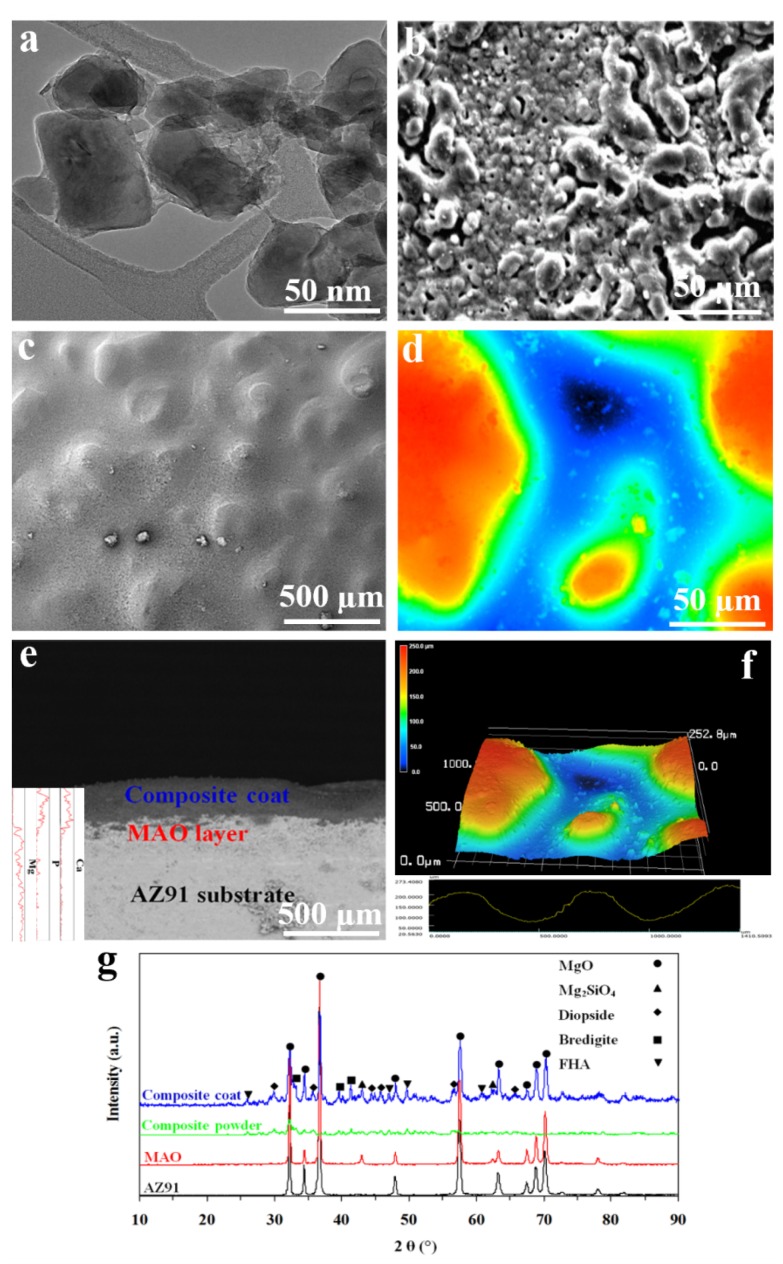
**Microstructure characterizations:** (**a**) TEM image of composite nanoparticles showing their size which was 50 ± 20 nm; (**b**) SEM image of a MAO treated AZ91 sample showing its rough and porous structure; (**c**–**f**) SEM (**c**,**e**) and LSM (**d**,**f**) images from top (**c**,**d**) and cross-section (**e**,**f**) of nanocomposite coated samples showing that the coating could effectively cover the pores of the MAO layer, the surface of nanocomposite coating was rough and porous, and the thickness of the MAO layer and nanocomposite coating was 100 and 250 µm, respectively. Also, the line-scan analysis of the cross-sectional SEM image confirmed that the nanocomposite coating mainly consists of Ca, P, and Mg elements. The intensity of Ca and P gradually decreased from nanocomposite coat to substrate, while Mg had an opposite trend (**e**); (**g**) XRD patterns of AZ91, MAO, and nanocomposite coated samples showing that AZ91 substrate had peaks related to Mg; MAO treated AZ91 had MgO and Mg_2_SiO_4_ peaks, and nanocomposite coating had peaks related to diopside, bredigite, and FHA.

The results of TEM imaging showed that our composite nanoparticles had a size of 50 ± 20 nm **(**Figure 3a). Following MAO treatment, a porous and rough surface was formed on AZ91 samples (Figure 3b). The MAO layer has been composed of one external porous layer and one internal compact barrier layer. The external layer is coarse with many microholes which have been formed due to the oxygen bubbles released during the growth process as well as the thermal stress as a result of the rapid solidification of the molten oxide in the relatively cooling electrolyte. However, the internal layer has been attached to the Mg substrate, is compact and uniform, and can act as a barrier to block the exposure of corrosive solutions to the Mg substrate [48]. The compact internal layer can insulate the substrate from the corrosive electrolyte ions while the external layer can absorb more corrosive electrolytes and therefore reduce the corrosion resistance of the Mg alloy substrate. Hence, the external layer of the MAO coating should be sealed by another coating layer. In addition, an MAO layer with a porous and rough surface can offer sites for our composite nanoparticles to be settled in. We then coated our MAO treated AZ91 with composite nanoparticles using the EPD process. The nanocomposite coating could adequately cover the pores of the MAO layer; this can then prevent the MAO layer from being directly exposed to the corrosive solutions. Similar to the MAO layer, the surface of our nanocomposite coating was rough and porous (Figure 3c,d). Using LSM, the surface roughness of AZ91 obtained 5 ± 3 µm; however, following coating with MAO and composite, the surface roughness increased to 12 ± 8, and 150 ± 80 µm, respectively. This can promote bone–implant integration since previous research has suggested that a rough and porous surface can encourage cell attachment and bone in-growth, which can enhance the anchorage of the implant to the bone [36]. Also, using SEM, the thickness of the MAO layer and nanocomposite coat obtained 100 and 250 µm, respectively (Figure 3e,f). The line-scan analysis of the cross-sectional SEM image confirmed that the nanocomposite coat mainly consists of Ca, P, and Mg elements. The intensity of Ca and P gradually decreased from nanocomposite coat to substrate, while Mg had an opposite trend (Figure 3e).

In the XRD pattern of the AZ91 substrate, Mg peaks were detected. When MAO treated AZ91 was tested with XRD, MgO, and Mg_2_SiO_4_ peaks were detected. MgO is formed by dissolving Mg^2+^ outward from the substrate and the oxidized oxygen O^2−^ inward from electrolyte according to reaction (3):

Mg^2+^ + O^2−^ → MgO (3)

Mg_2_SiO_4_ peaks indicate the existence of the anion SiO_3_^2−^. In an aqueous solution, the silicate is transformed into Si(OH)_4_ by hydroxylation. The water-assisted formation of Si(OH)_4_, which has 4 silanol groups (Si-OH) forms siloxane groups (i.e., Si–O–Si) and SiO_2_ during the strong electrical field and high-temperature anodization based on the reactions shown below (4) and (5) [49]:

4H_2_O + SiO_3_^2−^ → Si(OH)_4_ + 4OH^−^ (4)

Si(OH)_4_ + Si(OH)_4_ + …. → XSiO_2_ + yH_2_O (5)

At high temperatures, both SiO_2_ and MgO are present in the fused state [49].

However, during the interval stops of anodization sparking and micro-arcing, and by the cooling effect of the electrolyte, the fused state SiO_2_ and MgO forms Mg_2_SiO_4_ according to reaction (6):

SiO_2_ + 2MgO → Mg_2_SiO_4_ (6)

Mg_2_SiO_4_ is a bioactive ceramic [50], which can also have a protective effect on the AZ91 substrate [22].

The XRD patterns also confirmed the peaks related to diopside, bredigite, and FHA within the composite coat. The grain size of the composite coating was obtained to be approximately 25 nm according to the Williamson–Hall equation, confirming that our composite coating is a nanostructure material (Figure 3g).

### 3.2. Corrosion Tests

#### 3.2.1. Electrochemical Tests

**Figure 4:**
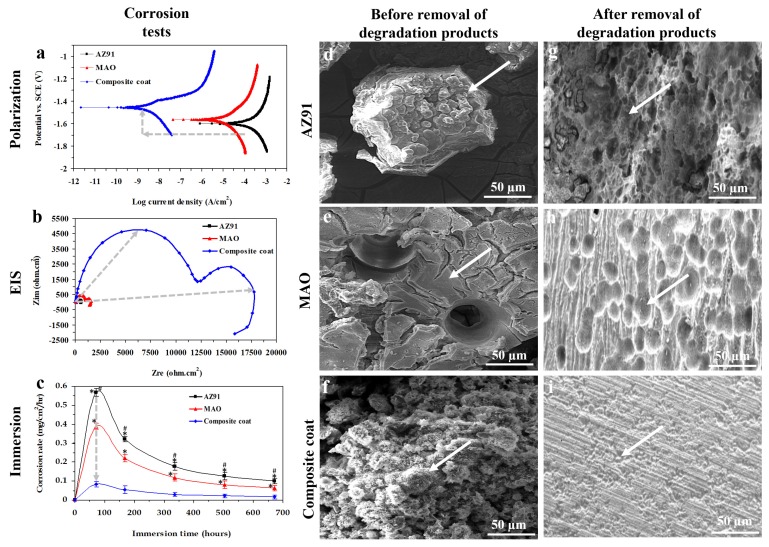
**Corrosion tests:** (**a**,**b**) Results of electrochemical corrosion tests include potentiodynamic polarization (**a**), EIS (**b**) and (**c**) immersion test showing that the corrosion rate of AZ91 Mg alloy substrate reduced following MAO and nanocomposite coating; (**d**–**i**) SEM images of AZ91 (**d**,**g**), MAO (**e**,**h**), and nanocomposite coated (**f**,**i**) samples after 672hr in SBF. Images have been taken before (**d**–**f**) and after (**g**–**i**) removal of degradation products. SEM images show that the cracks and pits formed on the AZ91 sample due to the corrosion were significantly more than MAO and nanocomposite coated samples. Furthermore, the surface of nanocomposite coated samples had been totally covered with cauliflower-like white particles. Significant differences: * *p* < 0.05: AZ91 vs. MAO or Composite coat, # *p* < 0.05: MAO vs. Composite coat.

The values of corrosion current density (i_corr_), and corrosion potential (E_corr_) derived from the potentiodynamic polarization curves (Figure 4a) showed that AZ91 sample has a 63,100 nA/cm^2^ i_corr_; This value decreased to 53,700, and 1.99 nA/cm^2^ for MAO and nanocomposite coated samples, respectively. The polarization test also recorded an increase in E_corr_ from −1.60 V to −1.56 and −1.45 V for AZ91, MAO, and nanocomposite coated samples, respectively. In general, a decrease in i_corr_ and an increase in E_corr_ is an indication of improvement in corrosion resistance [51]. EIS Nyquist plots showed that the Zim/Zre ratio of AZ91 increased with the MAO and nanocomposite coating, indicating an enhanced capacitive behavior for the solid/liquid interface. The MAO and nanocomposite coated samples showed larger capacitive loops in the EIS spectra than the AZ91 sample. Since a larger diameter loop represents better corrosion resistance [52], the EIS results confirm that the MAO and nanocomposite coating can improve the corrosion resistance of the AZ91 Mg alloy. Also, in the Nyquist plots, two capacitive loops and one inductive loop are seen for samples, similar to previously reported Nyquist plots of pure Mg [53]. The diameter of the loop in the high-frequency range is normally attributed to the charge transfer reaction, which is proportional to the transfer resistance value, i.e., R_t_. The larger the R_t_, the better is the corrosion resistance of coating [16]. From R_t_ value, the exchange-current density (j_0_) could also be calculated using Equation (7) [54]:

J_0_ = RT/nFR_t_ (7)

where n is the number of transferred charges, and F is Faraday constant. In Equation (7), j_0_ is in opposite proportion to R_t_, i.e., the higher the R_t_ is, the lower would be the corrosion rate [53]. Hence, charge transfer resistance could be utilized to assess the corrosion rate of the samples. This is because an increase in j_0_ should correspond to an increase in the corrosion rate. It can be deduced from EIS spectra that R_t_ of AZ91 samples increased from 137.6 Ω cm^2^ to 439.7 Ω cm^2^ and 5432.7 Ω cm^2^ for MAO and nanocomposite coated samples, suggesting that the nanocomposite coating is more corrosion resistant than AZ91, which is in good agreement with the results of polarization measurements (Figure 4b). Hence, the results of electrochemical tests reveal the increased corrosion resistance afforded by the nanocomposite coating. A delayed corrosion process is critical for a biodegradable implant, as the implant needs to maintain its mechanical functionality for a certain period before the bone defect is fully healed [55]. Therefore, the immersion tests can provide additional information regarding the corrosion rates of the AZ91, MAO, and nanocomposite coated samples for a longer time.

#### 3.2.2. Immersion Tests

The corrosion rate of the AZ91 sample obtained significantly higher than the MAO and nanocomposite coated samples (0.57 ± 0.02 vs. 0.39 ± 0.01 and 0.08 ± 0.01 mg/cm^2^/hr, respectively after 72 h immersion in the SBF) showing the effective protection provided by the MAO and nanocomposite coating (Figure 4c). Following the immersion test, local areas of the AZ91 surface were corroded, and many large cracks and pores were detected on the surface due to significant corrosion. Clusters of white particles had also been formed on the AZ91 surface (Figure 4d,g). The surface morphology of MAO treated AZ91 had too been corroded, and some pits and cracks were seen (Figure 4e,h). Comparing the surface morphology of samples following immersion showed that the density of cracks and pits formed on the AZ91 sample due to the corrosion were significantly higher than those formed on MAO and nanocomposite coated samples. It could be clearly seen that the MAO and nanocomposite coated samples had a more uniform and milder corrosion attack when compared to the AZ91 sample. The density of white particles formed on the surface of nanocomposite coated samples was also higher than MAO and AZ91 samples. In fact, the total surface of nanocomposite coated samples had been covered with cauliflower-like white particles (Figure 4f,i). Also, the degree of corrosion attack and formed white particles for the MAO sample was between AZ91 and nanocomposite coated samples (Figure 4e,h).

Due to the excellent castability, mechanical properties, corrosion resistance, and high maximum solubility of 12.7 wt.% in Mg, aluminum (Al) has been one of the most commonly used alloying elements for Mg-alloy systems in the early development stage of biodegradable orthopedic implants [56]. Mg–Al alloy systems such as AZ alloys, which were already processed for industrial applications, are currently available for further optimization, such as a surface coating as used in our study. In general, an increased Al content in Mg alloys enhances the ultimate tensile strength (UTS) and elongation up to 6 wt.% while reducing the corrosion rate by forming an aluminum oxide film [57]. Although Al is a well-known neurotoxicant linked with Alzheimer’s disease and dementia [58], researchers argue that the amount of Al released from such alloy systems with less than 5 wt.% Al is well below the weekly intake limits, and long-term in vivo studies have shown no direct detrimental effect [14,59]. AZ91 Mg alloy used in our study consists of 9 wt.% Al, i.e., higher than the threshold mentioned above (5 wt.%), however, using our nanocomposite coating system, the corrosion rate of AZ91 significantly reduced from 0.57 ± 0.02 to 0.08 ± 0.01 mg/cm^2^/hr which will also cause a significant reduction in Al release from our AZ91 substrate. Hence, our composite coated AZ91 offers a reduced corrosion rate as well as high mechanical properties.

The corrosion of Mg alloy proceeds by the following reactions:

Anodic reaction: Mg → Mg^2+^ + 2e^−^, (8)

Cathodic reaction: 2H_2_O + 2e^−^ → H_2_ + 2OH^−^, (9)

Total reaction: Mg _(s)_ + 2H_2_O _(aq)_ → Mg(OH)_2 (s)_ + H_2 (g)_, (10)

Mg(OH)_2 (s)_ + 2Cl^−^_(aq)_ → MgCl_2 (aq)_ + 2OH^−^_(aq)_. (11)

Following the immersion of an Mg alloy in the SBF, the electrolyte penetration followed by chemical dissolution results in the substrate to undergo rapid corrosion, and a magnesium hydroxide (Mg(OH)_2_) layer is then formed on its surface (reactions (8)–(10)). The deposition of Mg(OH)_2_ layer on the surface of the Mg alloy substrate can also act as a protective film that can prevent direct exposure of corrosion medium to Mg alloy. Mg(OH)_2_ would then react with chloride ions in the SBF and form the soluble MgCl_2_ (reaction (11)) [60]. This is the reason why our AZ91 Mg alloy sample had been subjected to a significant rate in the initial phase of immersion. Next, the formed corrosion product, which mainly contains Mg(OH)_2_, would thicken with immersion time, and the corrosion rate gradually decreases [61]. Although Mg(OH)_2_ forms on the surface of Mg alloy, it is too porous to protect the AZ91 substrate from corrosion effectively. Hence, continuous corrosion happens on Mg. This corrosion trend, i.e., fast corrosion initially followed by slow corrosion as time evolves, is also supported by our results (Figure 4c). However, our MAO and nanocomposite coating could act as an effective barrier to protect the AZ91 substrate from corrosion. Ca^2+^ ions and PO_4_^3−^ groups from the SBF and Mg^2+^ ions released from the AZ91 substrate also took part in the surface reaction of samples and form a calcium phosphate layer such as Ca_3_Mg_3_(PO_4_)_4_ on the sample (reaction (12)) which has a cauliflower-like structure [62]. Our SEM results also confirmed the formation of a layer with a cauliflower-like structure on the samples (Figure 4f). The formation of this phosphate coating can further protect the substrate from fast corrosion [63]. Therefore, a plateau in corrosion rates of AZ91, MAO, and nanocomposite coated samples were observed at the last stage of our immersion test, i.e., from 336 h to 672 h (Figure 4c).

Mg^2+^ + Ca^2+^ + PO_4_^3−^ → Ca_3_Mg_3_(PO_4_)_4_. (12)

### 3.3. In Vivo Animal Study

**Figure 5:**
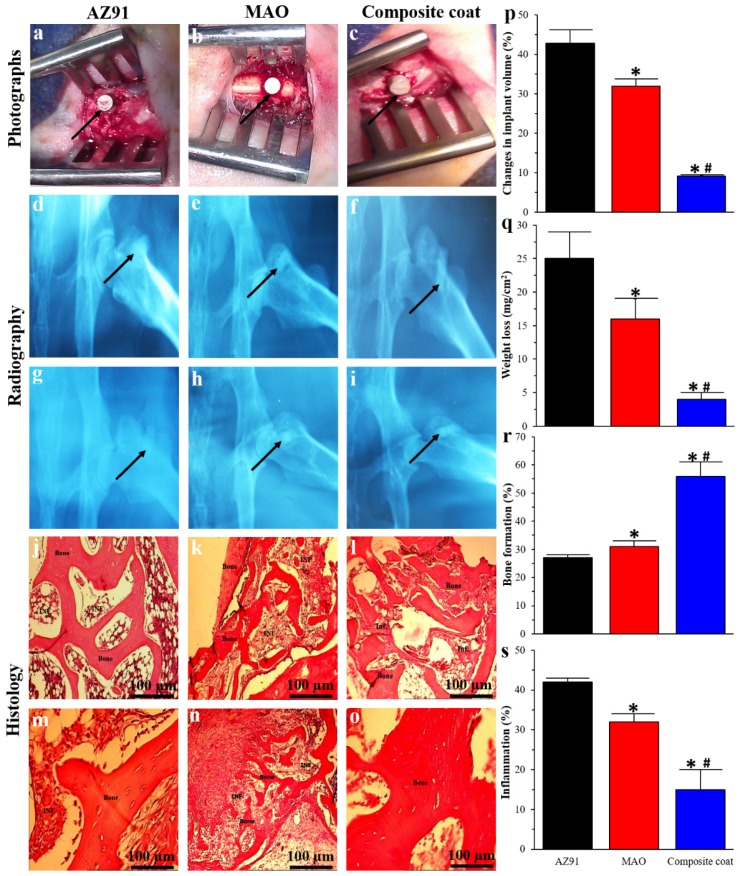
In vivo animal study: Surgical photos (**a**–**c**), radiography images (**d**–**i**), and histological images (**i**–**o**) of AZ91 (**a**,**d**,**g**,**j**,**m**), MAO (**b**,**e**,**h**,**k**,**n**), and nanocomposite coated (**c**,**f**,**I**,**l**,**o**) samples. Radiography images have been taken 2 weeks (**d**–**f**), and 2 months (**g**–**i**) after implantation. Black arrows in radiography images show the implants. Histological images have been presented in low (**j**–**l**) and high (**m**–**o**) magnification; Change in implant volume (**p**), weight loss (**q**), percentage of bone formation (**r**), and inflammation (**s**) for AZ91, MAO, and nanocomposite samples. Significant differences: * *p* < 0.05: AZ91 vs. MAO or Composite coat, # *p* < 0.05: MAO vs. Composite coat.

Following implantation, animals did not exhibit any sign of moribund/lethargic, distress, or local infections. Also, a normal wound healing was reported post-operation. In radiography images, AZ91 implants showed the highest hydrogen bubbles formation at the beginning, followed by a reduction over time. However, hydrogen bubbles formation reduced when MAO implants were used, and almost no hydrogen bubbles were seen around the nanocomposite coated implants (Figure 5d–i). Similarly, previous research has also shown that hydrogen bubbles are found around the Mg implants, which are then disappeared after 2–3 weeks [17,64]. In the first two weeks post-implantation, the rate of hydrogen-evolution from Mg implants is quicker than the hydrogen absorption rate. Over time, the corrosion rate reduces because of the formation of Mg(OH)_2_ and other corrosion products such as Ca_3_Mg_3_(PO_4_)_4_ [65]. Hence, the hydrogen bubbles around implants reduced from two weeks to two months. When the volume and weight of explanted implants were measured, the change in volume and weight of nanocomposite coated implants were significantly lower compared to AZ91 and MAO treated implants (volume change: 9.1 ± 0.2% vs. 42.8 ± 3.3% and 32 ± 1.7%; weight change: 4 ± 1 vs. 25 ± 4, and 16 ± 3 mg/cm^2^) showing the reduced in vivo corrosion of AZ91 implants when coated with our composite nanoparticles. Using histology, we observed new bone had been formed around the implants for all implants. However, compare to both AZ91 and MAO implants, nanocomposite coated implants showed the highest amount of new bone formation (56 ± 5% vs. 27 ± 1% and 31 ± 2%; Figure 5j–o,r). When inflammatory response in the tissue surrounding implants was compared, AZ91 had the highest inflammatory response; however, it decreased when MAO or composite coated implant was used (15 ± 3% vs. 42 ± 4% and 32 ± 4%; Figure 5j–o,s). This increase in bone formation and reduction in inflammatory response due to the composite coating on implants can be due to several reasons. One reason is a reduced corrosion rate and, therefore hydrogen-evolution since the coating decreases the direct contact of the implant with the body fluid. Furthermore, the production of hydrogen bubbles due to high corrosion of Mg alloys can prevent physiological bone reaction and callus formation [66], thereby resulting in a decrease in new bone formation and higher inflammation around the uncoated implants when compared to the coated ones.

Previous studies on biodegradable Mg alloys have shown that AZ91 Mg alloy corrodes at a rate of 1.1 mm/year [67], LAE442 at 2.8 mm/year [68], and WE43 at 3.9 mm/year [69]. Zinc, another biodegradable metallic implant, corrodes at a rate of 0.2 mm/year, which is a critically low corrosion rate for satisfactory biodegradable cardiovascular stents, although zinc corrodes more quickly after 3 months and should be removed away from the artery [70,71]. The corrosion rate for the AZ91 Mg alloy in our study obtained 1.3 mm/year, which is similar to the value reported for AZ91, i.e., 1.1 mm/year [72]. However, following MAO treatment and composite coating, the corrosion rate of AZ91 significantly reduced to 1.2 and 0.00005 mm/year, respectively, which is considerably lower than the corrosion rate of widely studied Mg alloys such as LAE442 or WE43 for orthopedic implant applications. This result demonstrates that our composite coat prepared by the MAO/EPD method is promising for applications with a strict requirement for corrosion rate. In addition to reducing the corrosion rate, our strategy will also give bioactivity, i.e., osteoproductivity to Mg, which makes it a suitable platform for bone implantation and regeneration. Although our nanocomposite coating has been applied on AZ91 Mg alloy, this surface coating material (i.e., diopside-bredigite-FHA) with its coating method (i.e., MAO/EPD) can also be utilized on other biodegradable Mg alloys where a reduced corrosion rate, as well as an enhanced bioactivity, are required.

## 4. Conclusions

A nanocomposite made of diopside, bredigite, and fluoridated hydroxyapatite bioceramics were successfully coated on a biodegradable AZ91 Mg alloy using micro-arc oxidation followed by an electrophoretic deposition method. Following coating, the corrosion rate of AZ91 reduced from 0.57 ± 0.02 to 0.08 ± 0.01 mg/cm^2^/hr, which resulted in a reduced hydrogen-evolution in vivo. Improved bone regeneration (27 ± 1% to 56 ± 5%) with a reduced inflammatory response (42 ± 4% to 15 ± 3%) was detected in the tissue surrounding the composite coated implant compared to the uncoated ones. Hence, our composite coating strategy can be used on biodegradable Mg bone implants, where a reduced corrosion rate and improved implant osteointegration are required. Our results will help shed not only new light on the possible development of Mg-based orthopedic implants, i.e., bone plates, screws, pins, and nails, but also provide guidelines for the development and surface coating of Mg-based porous scaffolds for bone tissue engineering.
